# Oxidative stress and Nrf2 expression in peripheral blood mononuclear cells derived from COPD patients: an observational longitudinal study

**DOI:** 10.1186/s12931-020-1292-7

**Published:** 2020-01-30

**Authors:** A. M. Fratta Pasini, C. Stranieri, M. Ferrari, U. Garbin, L. Cazzoletti, C. Mozzini, F. Spelta, D. Peserico, L. Cominacini

**Affiliations:** 1 Department of Medicine, Section of General Medicine and Atherothrombotic and Degenerative Diseases, University of Verona, Verona, Italy; 2 Department of Medicine, Unit of Respiratory Diseases, Verona, Italy; 3 Department of Diagnostics and Public Health, Unit of Epidemiology & Medical Statistics, University of Verona, Verona, Italy

**Keywords:** COPD progression, Oxidative stress, Nrf2/ARE gene expression, PBMCs

## Abstract

**Background:**

A persistent low inflammatory-oxidative status and the inadequacy of the antioxidant nuclear factor-E2-related factor 2 (Nrf2) have been implicated in chronic obstructive pulmonary disease (COPD) progression. Therefore this study was aimed to assess the association between lung function decline and oxidative-inflammatory markers and Nrf2 signaling pathway expression in peripheral blood mononuclear cells (PBMCs) over time.

**Methods:**

33 mild-moderate COPD outpatients (mean age 66.9 ± 6.9 years) were age-sex matched with 37 no-COPD subjects. A clinical evaluation, blood sampling tests and a spirometry were performed at baseline and after a mean follow-up of 49.7 ± 6.9 months.

**Results:**

In COPD, compared to no-COPD, we found a faster lung function decline at follow-up. Although similar prevalence of smoking, hypertension, diabetes and dyslipidemia, systemic markers of inflammation (hs-CRP and white blood cells, WBCs) and oxidative stress (8-isoprostane) were significantly increased in COPD at follow-up, while the antioxidant glutathione (GSH) was significantly reduced. Moreover the expression of Nrf2 and of Nrf2-related genes heme oxygenase (HO)-1 and glutamate-cysteine ligase catalytic (GCLC) subunit in PBMCS were significantly down-regulated in COPD at follow-up, whereas no changes were observed in no-COPD. The percent variation (Δ) of FEV_1_ detected after the follow-up in COPD patients was directly correlated with ΔNrf2 (r = 0.826 *p* < 0.001), ΔHO-1 (r = 0.820, p < 0.001) and ΔGCLC (r = 0.840, p < 0.001). Moreover ΔFEV_1_ was also directly correlated with ΔGSH (r = 0.595, *p* < 0.01) and inversely correlated with Δ8-iso (r = − 0.587, p < 0.01) and with baseline smoking history (r = − 0.39, *p* < 0.03). No correlation was found between ΔFEV_1_, ΔCRP and ΔWBCs. By means of hierarchical stepwise multiple linear regression, taking into account other baseline key factors related to FEV_1_, ΔNrf2, ΔHO-1and ΔGCLC were found to be significant predictors of ΔFEV_1_, explaining 89.5% of its variance.

**Conclusions:**

Although our results must be confirmed in larger trial they suggest that the down-regulation of Nrf2/ARE gene expression in PBMCs may be one of the determinants of FEV_1_ decline and of COPD progression. Therefore the future possibility to counteract Nrf2 decline in COPD patients may help in reducing the negative effects of the oxidative stress-induced progression of the disease.

## Background

Chronic obstructive pulmonary disease (COPD) is foreseen to become the sixth leading cause of disability and the third cause of death by 2020 [1, 2]. In spite of the fact that cigarette smoking is the major risk factor for COPD [3], the evidence that only a relatively small group of smokers develop COPD [4] and that cigarette smoking discontinuation only partially halts disease progression [5] has led to the suggestion that other elements may also be driving the disease. Compelling data suggest that oxidative stress and inflammation may have a key pathogenetic role in COPD beginning and evolution [6, 7]. In COPD patients, oxidative stress derives from reactive oxygen species (ROS) present in cigarette smoking per se and/or may be triggered by various inflammatory and immune stimuli in epithelial cells of the airways [7]. Incremented oxidative stress, in turn, strengthens pulmonary inflammation with subsequent recruitment and activation of immune cells into the lungs, and production of inflammatory mediators [7]. In this context, it has been reported that host ability to protect from oxidative stress by upregulating lung antioxidant defenses may be one of the crucial circumstances that dictates the severity and progression of COPD [8].

Nuclear factor-E2-related factor (Nrf2) is an emerging regulator of cellular resistance to oxidative stress. Nrf2 regulates the basal and induced expression of a series of antioxidant response element (ARE)-dependent genes as heme-oxygenase (HO)-1 and glutamate-cysteine ligase catalytic (GCLC) subunit which regulate the physiological and pathophysiologic outcomes of oxidant exposure [9]. In basal conditions, Nrf2-dependent transcription is blocked by its repressor Kelch-like ECH-associated protein 1; under conditions of cellular oxidative stress, Nrf2 moves to the nucleus and determines the expression of its target genes [9]. Several reports indicate a critical role for Nrf2 in counteracting lung diseases; in particular, previous studies have shown an increased risk of emphysema induced by cigarette smoking in Nrf2-deficient mice [10] and a reduction of Nrf2 expression in pulmonary macrophages of current smokers and COPD patients [11]. Furthermore the evidence that the pharmacological activation of Nrf2 can delay the progression of experimental emphysema, suggests that Nrf2 may play a pathogenetic role in lung diseases [12]. COPD is typically diagnosed late in the course of disease when the symptoms become clinically evident [13] and consequently very few studies have focused on oxidative stress in its early phases, potentially crucial for the subsequent evolution of airway damage. In this context, our group has formerly demonstrated that mild-moderate ex-smokers with COPD may be able to counteract oxidative stress by increasing the expression of Nrf2/ARE in peripheral blood mononuclear cells (PBMCs) [14]. These results are in line with the demonstration that some oxidative products of phospholipids stimulated the generation of ROS in PBMCs by triggering the activation of nicotinamide adenine dinucleotide phosphate oxidase [15] and that the increase of these oxidized compounds in PBMCs was associated with the activation of the Nrf2/ARE pathway in mild smokers compared to nonsmokers, whereas in heavy smokers the Nrf2/ARE expression was similar to no-smokers [15]. Therefore, this study was performed in COPD patients with mild-moderate bronchial obstruction compared to age, sex-matched no-COPD subjects and was aimed to evaluate over time: 1) the behavior of circulating oxidative-inflammatory markers and Nrf2/ARE expression in PBMCs; 2) the lung function decline and its relationship with the changes of circulating oxidative-inflammatory parameters and Nrf2/ARE expression in PBMCs.

## Methods

The study was approved by the Ethic Committee of the Azienda Ospedaliera Universitaria Integrata Verona (prot. n. 42052/2015), in agreement with the principles of the Declaration of Helsinki, and written informed consent was acquired from all the subjects before their enrollment.

### Study population and follow-up

Sample size was estimated based on the behavior of FEV_1_ decline (ml/year) in Global Initiative for Chronic Obstructive Lung Disease (GOLD) 1 and GOLD 2 stage COPD patients and of Nrf2 mRNA as previously reported [14, 16]. Adopting a level of significance of *p* = 0.05, a power of 80% and a minimum correlation of 0.40, we estimated the minimum sample size to be approximately 32 subjects [17]. At beginning of the study we enrolled 60 consecutive, mild-moderate (GOLD 1, *n* = 29; GOLD 2, *n* = 31;) COPD patients referring to Respiratory Medicine Outpatient Clinic of our Institution. The GOLD guideline was used to make the diagnosis and to grade COPD severity [18]. The other group (*n* = 71) comprised age-sex-matched no-COPD subjects randomly selected from the general population [19]. Principal necessary conditions for the enrollment of both groups were lack of infectious or acute/chronic inflammatory diseases, malignancy, absence of acute/chronic renal failure and hepatic failure. No COPD subjects were using supplemental oxygen, oral glucocorticoids, and antibiotics. Inhaled corticosteroid and bronchodilator agents were administered following guideline [18, 19]. A clinical evaluation, blood sampling tests and a spirometry was performed at baseline and after a mean follow-up of at least 40 months.

### Pulmonary function test

Forced expiratory volume in 1st second (FEV_1_) and FEV_1_/forced vital capacity (FVC) were measured using a water-sealed spirometer (Biomedin, Padua, Italy). Lung function values were expressed as a percentage of predicted values, and the lower limit of normal low limit of normality for the FEV_1_/FVC was calculated according to Quanjer [20].

### Blood samples and PBMC isolation

Venous blood samples were obtained from each subject after 12 h fasting and drawn into pyrogen-free blood collection tubes. Several aliquots of plasma were placed into sterile 1 mL screw-capped polypropylene vials containing the phenolic antioxidant 2,6-di-tert-butyl-4-methylphenol (10 mM, Sigma-Aldrich Co., St Louis, MO, USA) to avoid lipid peroxidation and stored at − 80 °C. The samples were frozen and thawed only once. PBMCs were isolated as previously described [21]. High sensitivity C-reactive protein (CRP) was evaluated using a commercially available high-sensitivity turbidimetric method (Syncron-PCR; Beckman Coulter, Brea, CA, USA). Plasma 8-isoprostane (8-iso) was measured by means of Cayman’s 8-iso ELISA kit following the manufacturer’s indications. Total glutathione (GSH) was measured by means of Abcam’s GSH/GSSG ratio detection kit.

### RNA isolation and quantitative real-time polymerase chain reaction

Total RNA was isolated with RNEasy Mini Kit (Qiagen, Hilden, Germany). The concentration and quality of RNA were evaluated using the RNA 6000 Nano LabChip Kit (Agilent 2100 Bioer, Agilent Technologies Inc., Santa Clara, CA, USA). Reverse transcription of total RNA was carried out using IScript cDNA Synthesis Kit (Bio-Rad, Hercules, CA, USA) according to the manufacturer’s recommendations. The relative mRNA expression levels of Nrf2, HO-1 and GCLC were performed in triplicate using the QuantiTect Primer Assay and QuantiTect SYBR Green PCR Kit (Qiagen) on the MyiQ Thermal Cycler (Bio-Rad). QuantiTect Hs-ACTB Assay (Qiagen) was used as normalizer. Normalized gene expression levels are given as the ratio between the mean value for the target gene and that for the beta-actin in each sample.

### Statistical analysis

Continuous variables are expressed as mean ± SD values. Differences between the groups were analyzed by two-tailed paired and unpaired Student’s t-test. Categorical variables were compared using the Chi squared test**.** Pearson’s correlations were used to test the relationship between the variables. A hierarchical stepwise multiple linear regression model was used to evaluate the joint effect of independent variables percent variation (Δ) on ΔFEV_1_ (% predicted, dependent variable). ΔNrf2 mRNA, ΔHO-1 mRNA, ΔGCLC mRNA, Δ8-iso and ΔGSH were the independent variables considered. In the model, other baseline key factors related to change in FEV_1_ including baseline FEV_1_, age, sex, smoking status, smoking history (pack year), BMI and follow-up time between FEV_1_ measurements were taken into consideration. To exclude model overfitting, the estimated regression equation of SPSS to calculate the predicted value of R-squared was used. Missing data were handled via complete case analysis as previously suggested [22]. We also assessed the association between the dependent variable FEV_1_ and the independent variables Nrf2 mRNA, HO-1 mRNA, GCLC mRNA, 8-iso and GSH, a 2-level random intercept linear model was fitted to the data, with level 1 units (measurements/visits) nested into level 2 units (subjects). The between-subject variability was modeled as a random effect, i.e. as a random-intercept term at the subject level. The Huber/White sandwich estimate of variance was used at the highest level (subjects) in the mixed model. The model also adjusted for age, sex, smoking status, smoking history (pack year), BMI and follow-up time. A *p* value less than 0.05 was considered significant. Statistical analysis of the data was conducted using SPSS version 20.0 (IBM Corporation, Armonk, NY, USA).

## Results

### Lung function, clinical and laboratory characteristics

**Fig. 1:**
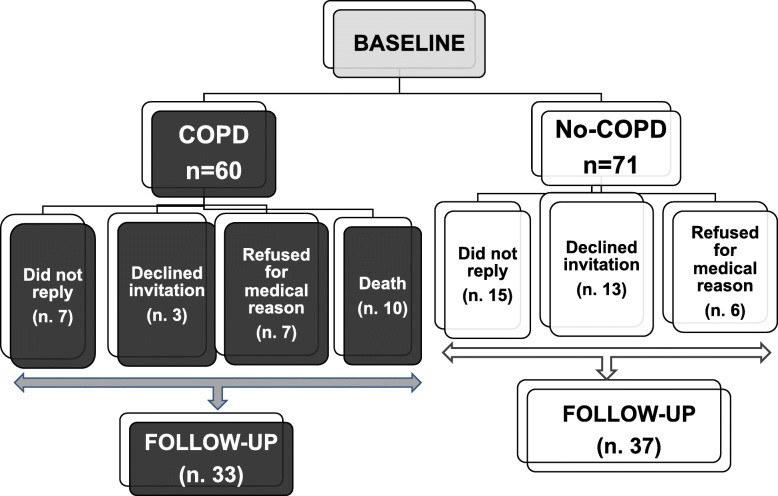
Detailed diagram of participant flow and group allocation

**Fig. 2:**
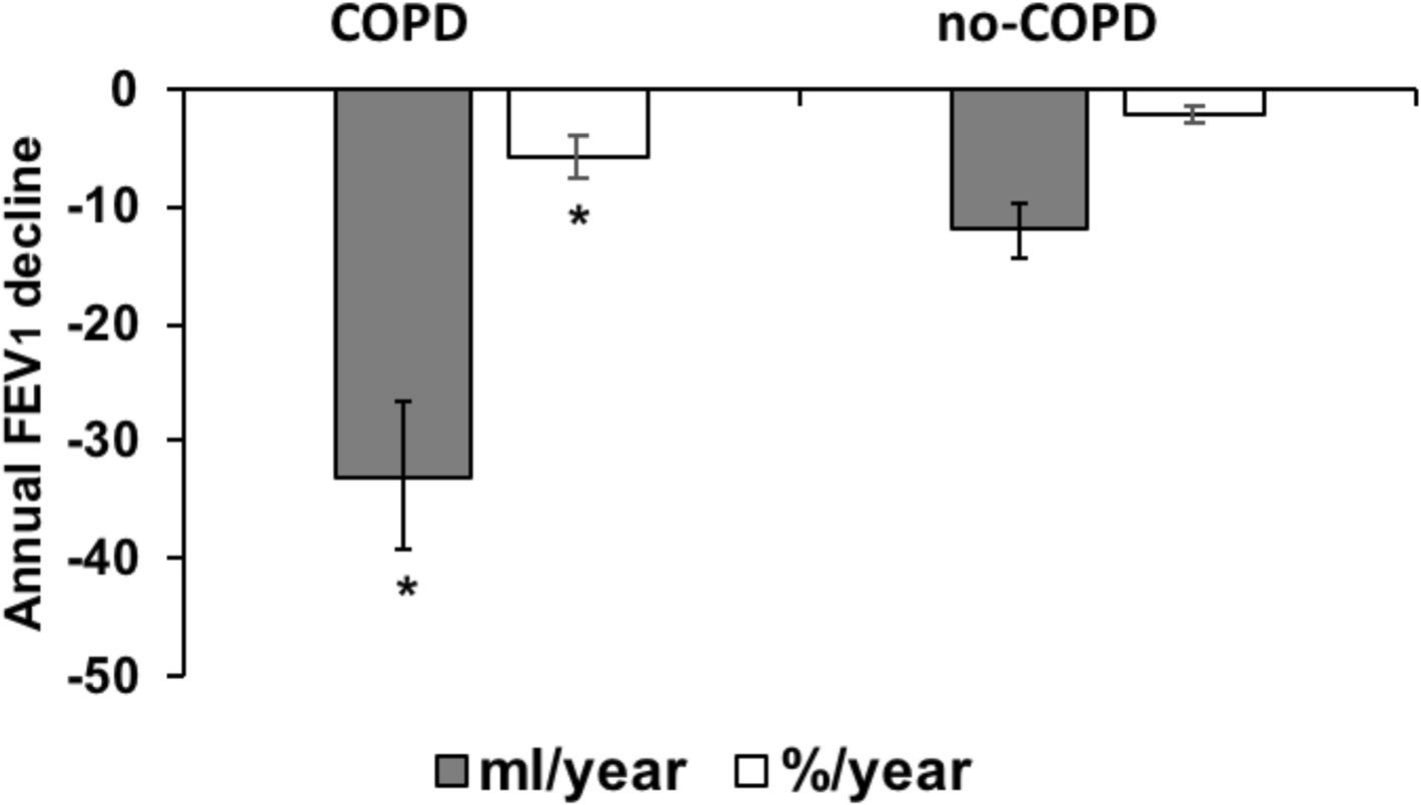
Annual lung function decline in COPD and no-COPD groups after the period of follow-up. Data are expressed as mean ± SD and represent FEV_1_ annual variations (in ml/year and %/ year) between baseline and follow-up values. *p < 0.001 vs no-COPD. Abbreviations: COPD, chronic obstructive pulmonary disease; FEV_1_: Forced expiratory volume in 1st second

At baseline 131 subjects (60 mild-moderate COPD and 71 no-COPD) were selected. In agreement with the inclusion criteria, basal FEV_1_ (actual and % of predicted) and FEV_1_/FVC (actual and % of predicted) resulted significantly lower in COPD than in no-COPD subjects (data not shown). After a period of at least 40 months (mean 49.7 ± 6.9 months) of the 131 subjects enrolled in the first phase of the study, 7 COPD and 15 no-COPD did not reply, 3 COPD and 13 no-COPD declined invitation, 7 COPD and 6 no-COPD refused for supervening medical reasons. Furthermore 10 COPD subjects were meanwhile dead. Figure 1 shows a detailed diagram of participant flow and group allocation**.** Of the 61 missing data, 38 were not related to medical issues or death, and reasonably lost at random, while 23 were missed not at random. Lung function, anthropometric, clinical and metabolic parameters of drop-out subjects were similar to those of the subjects participating to the follow-up. Therefore, 70 subjects (37 no-COPD and 33 COPD patients) participated to both baseline and follow-up phases. GOLD stage and lung function for both groups are reported in Table 1. Not unexpectedly, GOLD stage change was different in no-COPD compared to COPD group after the follow-up period. In particular only few subjects resulted slightly obstructed (GOLD 1) in no-COPD group at follow-up. In the COPD group the number of patients classified as GOLD 1 and GOLD 2 at baseline was significantly reduced after the follow-up period; therefore, some subjects were classified as GOLD 3 and GOLD 4 after the period of follow-up. Accordingly, FEV_1_ and FEV_1_/FVC at follow-up were significantly lower than at baseline in COPD patients, whereas no differences were observed in no-COPD group. As shown in Fig. 2, lung function decline (expressed as annual FEV_1_ variation between baseline and follow-up values) was much greater in COPD patients than in no-COPD subjects (*p* < 0.01). Medical history, clinical and metabolic characteristics of both groups of subjects at baseline and at follow-up are shown in Table 1**.** Concerning cardiovascular risk factors hypertension, diabetes and dyslipidemia were similar in COPD and no-COPD subjects at baseline. On the contrary, the two groups differed for smoking habit: in particular the number of no smokers was significantly higher in no-COPD than in COPD subjects, whereas the number of past-smokers was higher in COPD group. As shown in Table 1, the number of current smokers was small in both groups: after the period of follow-up, it reduced from 5 to 2 in no-COPD and from 6 to 4 in COPD. Accordingly, there was an increment of the number of past smokers in both groups. Moreover basal and anthropometric characteristics, lipid profile, plasma glucose, systolic and diastolic blood pressure were similar in both groups both at baseline and at follow-up.

**Table 1:** GOLD stage, lung function, medical history, clinical and metabolic parameters of both groups of subjects at baseline and at follow-up

	No-COPD Baseline	No-COPD Follow-up	COPD Baseline	COPD Follow-up
GOLD 0 *n*. (%)	37 (100)	30 (82.1)	0	0
GOLD 1 *n*. (%)	0	7 (18.9)	17 (51.5)*	12 (36.4)**
GOLD 2 *n*. (%)	0	0	16 (49.5)	11 (33.3)**
GOLD 3 *n*. (%)	0	0	0	8 (24.2)
GOLD 4 *n*. (%)	0	0	0	2 (6.1)
FEV1 (L)	2.9 ± 0.5	2.9 ± 0.6	1.7 ± 0.3*	1.4 ± 0.7*,**
FEV1 (% of predicted)	109.5 ± 18.2	111.0 ± 18.3	71.5 ± 25.1*	66.4 ± 26.4**
FEV1/FVC actual (%)	76.4 ± 4.3	73.9 ± 5.9	55.4 ± 12.1*	51.3 ± 13.9*
FEV1/FVC (% of predicted)	98.8 ± 8.9	97.3 ± 9.4	73.6 ± 16.3*	68.9 ± 18.1*
Age (years)	68.9 ± 6.6	72.2 ± 6.8	70.1 ± 7.7	73.5 ± 7.9
Sex (M/F)	27/10	–	24/9	–
No-smokers *n*. (%)	21 (56.8)	21 (56.8)	3 (9.1)*	3 (9.1)*
Past-smokers *n*. (%)	11 (29.7)	14 (37.8)	24 (72.7)*	26 (78.8)*
Smokers n. (%)	5 (13.5)	2 (5.4)**	6 (18.2)	4 (12.1)*
Hypertension *n*. (%)	25 (67.6)	–	25 (83.3)	–
Type 2 Diabetes *n*. (%)	4 (10.8)	–	5 (16.6)	–
Dyslipidemia *n*. (%)	32 (86.5)	–	26 (86.6)	–
Antihypertensive drug use *n*. (%)	21 (56.7)	22 (59.4)	23 (76.6)	21 (70.0)
Antidiabetic drug use *n*. (%)	1 (2.7)	3 (8.1)	4 (13.3)	5 (16.6)
Statin use *n*. (%)	12 (32.4)	11 (29.7)	13 (43.3)	11 (33.3)
BMI (kg/m^2^)	26.3 ± 3.7	26.5 ± 3.9	26.6 ± 3.9	26.9 ± 4.7
SBP (mmHg)	137.8 ± 17.2	142.0 ± 15.0	133.1 ± 14.9	140.4 ± 16.2
DBP (mmHg)	84.4 ± 9.8	82.2 ± 7.7	82.4 ± 9,1	83.7 ± 7.5
Total cholesterol (mmol/L)	5.6 ± 1.0	5.0 ± 1.1	5.3 ± 1.2	5.1 ± 1.0
LDL cholesterol (mmol/L)	3.6 ± 0.9	3.2 ± 0.7	3.1 ± 1.0	2.9 ± 0.8
HDL cholesterol (mmol/L)	1.5 ± 0.4	1.4 ± 0.5	1.4 ± 0.4	1.4 ± 0.5
Triglycerides (mmol/L)	1.3 ± 0.4	1.4 ± 0.5	1.4 ± 0.4	1.1 ± 0.4
Plasma glucose (mmol/L)	5.5 ± 1.3	5.6 ± 1.7	5.7 ± 0.6	5.5 ± 0.9

Note: Continuous variables are expressed as mean ± SD; *p < 0.001 vs no-COPD baseline or follow-up; ** *p* < 0.01 vs baseline

Legend: *GOLD* Global initiative for Chronic Obstructive Lung Disease, *FEV1* Forced expiratory volume in 1st second, *FVC* forced vital capacity, *BMI* body mass index, *SBP* systolic blood pressure, *DBP* diastolic blood pressure

### Circulating markers of inflammation and oxidative stress in both groups of subjects at baseline and at follow-up

**Fig. 3:**
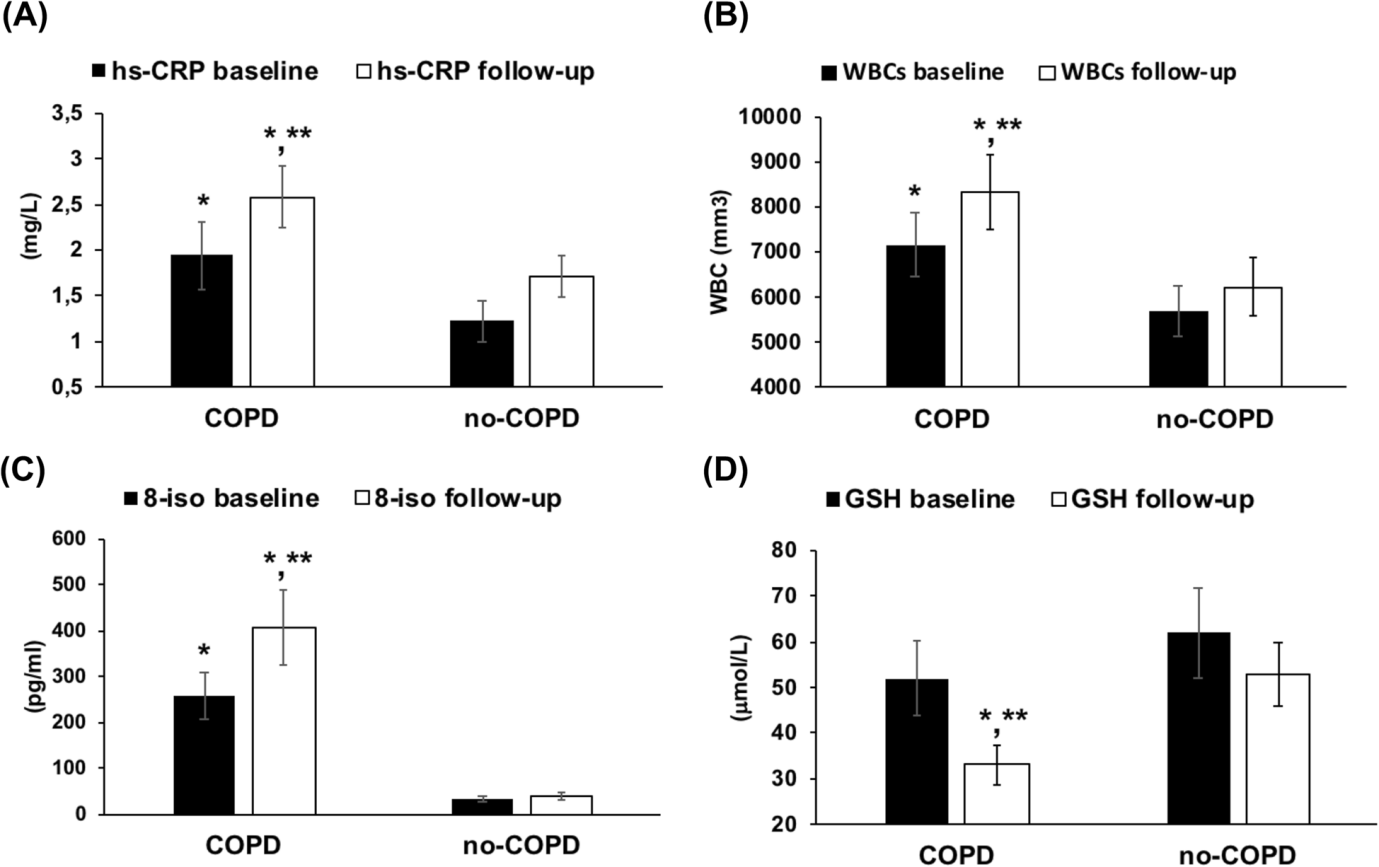
Circulating markers of inflammation and oxidative stress in COPD and no-COPD groups at baseline and at follow-up. **a** High-sensitivity C-reactive protein (CRP) plasma concentrations. **b** White blood cells (WBCs) count. **c** 8-isoprostane (8-iso) plasma concentrations. **d** Glutathione (GSH) plasma concentrations. Data are expressed as mean ± SD. *p < 0.01 vs no-COPD baseline or follow-up. **p < 0.01 vs baseline

Plasma CRP concentrations were higher (*p* < 0.01) in COPD than in no-COPD subjects at baseline. After the follow-up, CRP persisted to be higher in COPD patients than in controls (*p* < 0.01) and the values were even higher than the values detected at baseline (*p* < 0.01), (Fig. 3a). White blood cells (WBCs), even if in the normal range, were significantly higher in COPD than in no-COPD at baseline (p < 0.01). After the follow-up there was a further significant increment of WBCs in COPD when compared with the baseline values (*p* < 0.01) (Fig. 3b).

As for 8-iso plasma concentrations the results of this study show that at baseline 8-iso values were significantly more elevated in COPD than in no-COPD subjects (*p* < 0.001). After the follow-up, 8-iso concentrations continued to be higher in COPD than in no-COPD and moreover 8-iso values resulted further increased than those observed at baseline (*p* < 0.01), (Fig. 3c).

Circulating GSH concentrations were similar in COPD and in no-COPD subjects at baseline. After the period of follow-up, GSH values were significantly reduced in COPD compared to both no-COPD subjects (*p* < 0.01) and to the corresponding values measured at baseline (p < 0.01), (Fig. 3d).

### Nrf2/ARE signaling pathway expression in PBMCs derived from COPD and no-COPD subjects at baseline and at follow-up

**Fig. 4:**
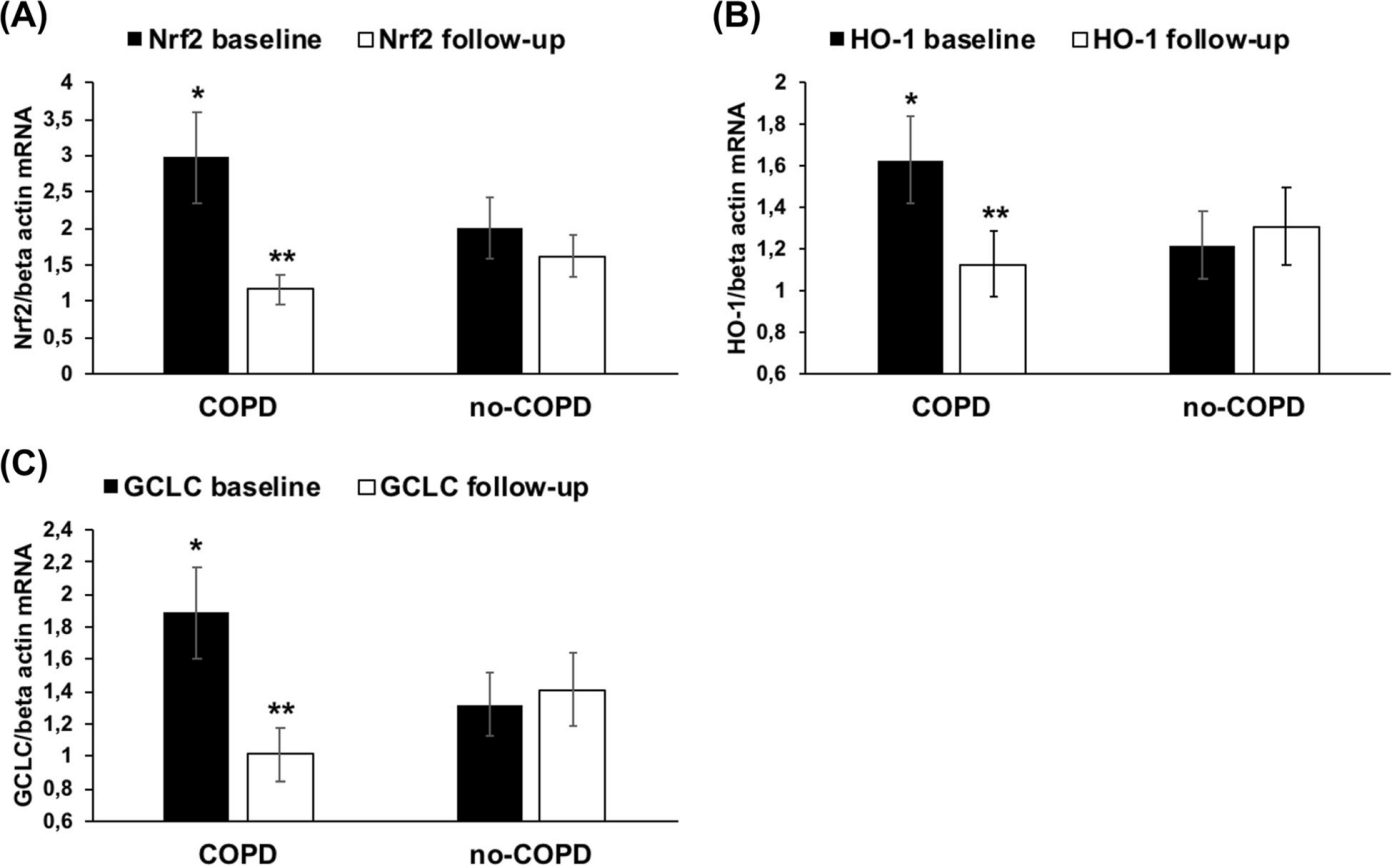
Nrf2/ARE mRNA expression in PBMCs derived from both groups at baseline and at follow-up. mRNA was analyzed by quantitative real-time PCR; normalized gene expression levels are given as the ratio between the mean value for the target gene and β-actin in each sample. Data are expressed as mean ± SD; *p < 0.01 versus no-COPD. **p < 0.01 vs baseline. Abbreviations: GCLC, glutamate-cysteine ligase catalytic; HO-1, heme oxygenase-1; mRNA, messenger RNA; Nrf2, nuclear factor-E2-related factor 2; PBMCs, peripheral blood mononuclear cells; PCR, polymerase chain reaction.** a** Nrf2 mRNA expression. **b** HO-1 mRNA expression. **c** GCLC mRNA expression

We also evaluated the mRNA expression of Nrf2 and of its correlated genes HO-1 and GCLC in both groups of subjects. As shown in Fig. 4(a-c), at baseline the expression of Nrf2, HO-1 and GCLC was significantly (*p* < 0.01) up-regulated in COPD compared to no-COPD group. After the follow-up there was a significant down-regulation of Nrf2, HO-1 and GCLC expression in COPD patients (p < 0.01), while in no-COPD subjects their expression did not change.

### Relationship between lung function and Nrf2/ARE gene expression and circulating markers of oxidative stress and inflammation in COPD group

**Fig. 5:**
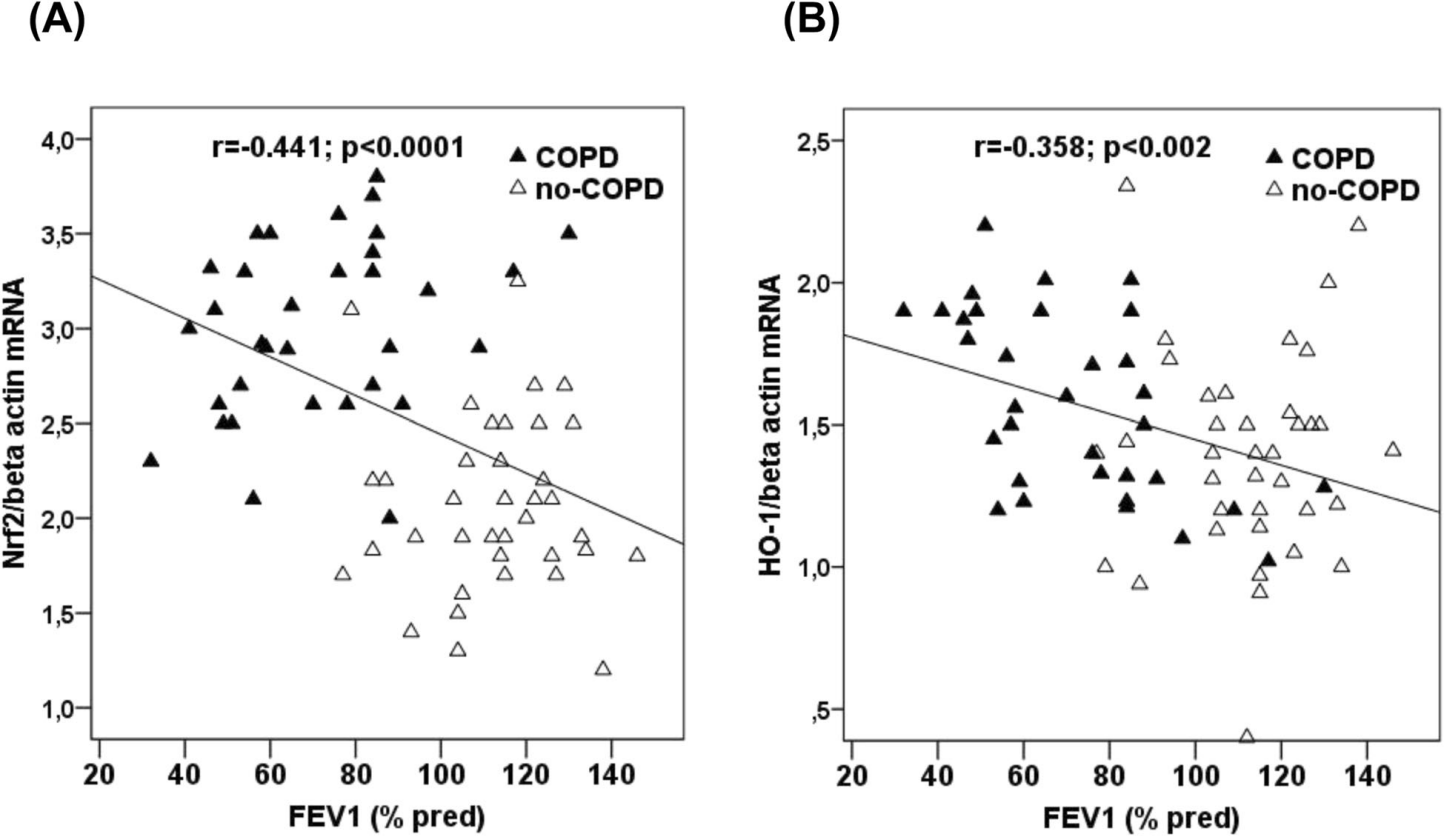
Correlations between FEV_1_ percent predicted (% pred) and Nrf2 and HO-1 gene expression in both groups of subjects at baseline. **a** Correlation between FEV_1_ and Nrf2 mRNA expression in PBMCs. **b** Correlation between FEV_1_ and HO-1 mRNA expression in PBMCs. Abbreviations: HO-1, heme oxygenase-1; mRNA, messenger RNA; Nrf2, nuclear factor-E2-related factor 2; PBMCs, peripheral blood mononuclear cells

At baseline FEV_1_ was inversely correlated with Nrf2 mRNA (r = − 0.441, *p* < 0.001) (Fig. 5a) and HO-1 mRNA (r = − 0.358, *p* < 0.002) (Fig. 5b) in all the subjects. After the period of follow-up, Nrf2 and HO-1 mRNA were no longer correlated with FEV_1_.

**Fig. 6:**
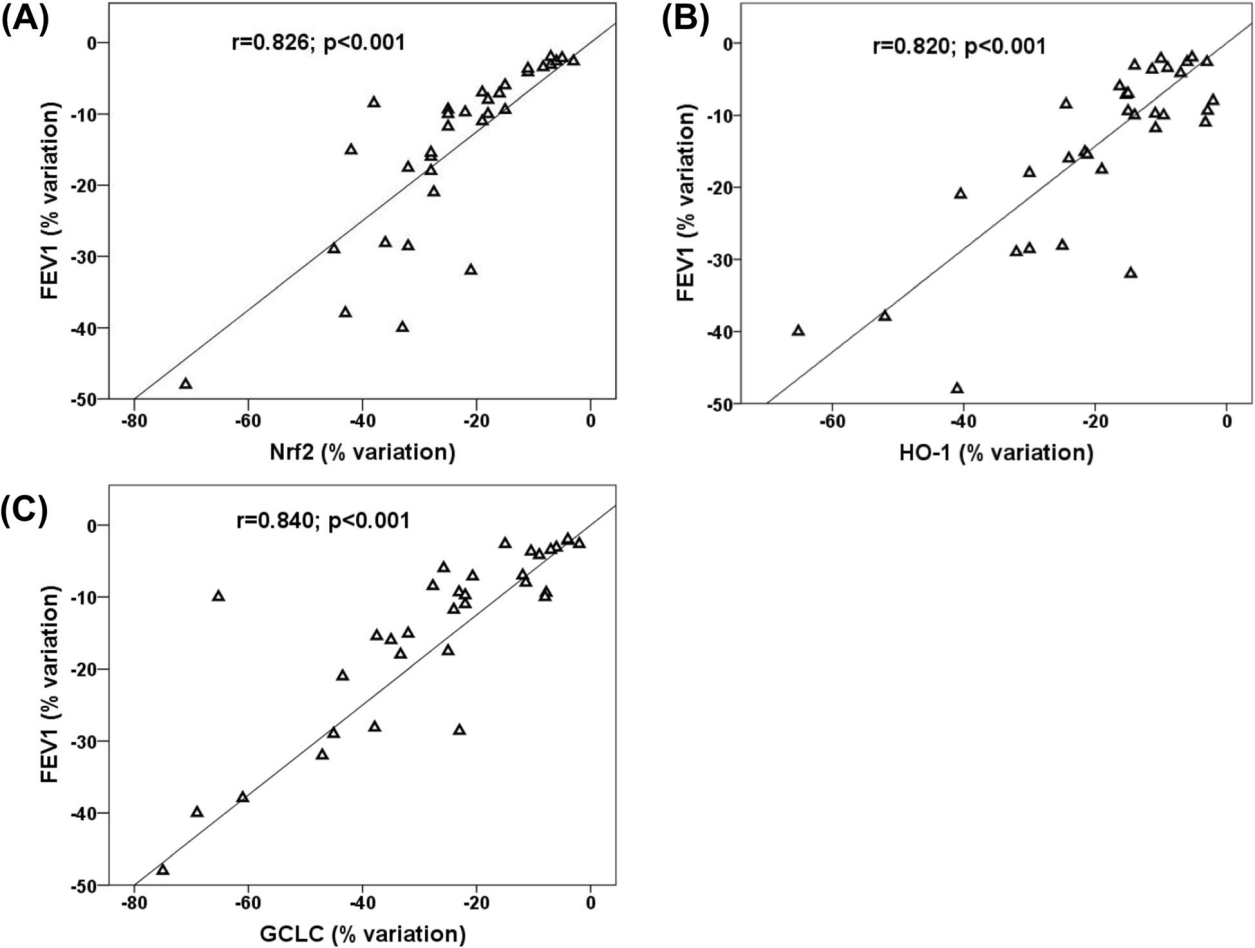
Correlations between FEV_1_ percent variation (Δ) and Δ Nrf2/ARE gene expression in COPD patients. **a** Correlation between FEV_1_ and Nrf2 mRNA expression in PBMCs. **b** Correlation between FEV_1_ and HO-1 mRNA expression in PBMCs. **c** Correlation between FEV_1_ and GCLC mRNA expression in PBMCs. Abbreviations: GCLC, glutamate-cysteine ligase catalytic; HO-1, heme oxygenase-1; mRNA, messenger RNA; Nrf2, nuclear factor-E2-related factor 2; PBMCs, peripheral blood mononuclear cells

ΔFEV_1_ detected after the follow-up in COPD patients was directly correlated with ΔNrf2 (r = 0.826, p < 0.001) (Fig. 6a), ΔHO-1 (r = 0.820, p < 0.001), (Fig. 6b), ΔGCLC (r = 0.840, p < 0.001), (Fig. 6c). Moreover ΔFEV_1_ was also directly correlated with ΔGSH (r = 0.595, *p* < 0.01, data not shown) and inversely correlated with Δ8-iso (r = − 0.587, p < 0.01, data not shown). Furthermore among the further baseline key factors which may influence ΔFEV_1_ (baseline FEV_1_, age, sex, smoking status, pack year, BMI and follow-up time between FEV_1_ measurement) only pack year resulted inversely correlated with ΔFEV_1_ (r = − 0.39. *p* < 0.03, data not shown). Finally no correlation was found between ΔFEV_1_, ΔCRP and ΔWBCs.

We then performed a model of hierarchical stepwise multiple linear regression in order to evaluate the combined effect of independent variables on FEV_1_ decline taking into account some baseline key factors (baseline FEV_1_, age, sex, smoking status, pack year, BMI and follow-up time) which may be related to FEV_1_. Our results show that Δ8-iso, ΔGSH plasma concentrations and baseline pack year were no longer correlated with ΔFEV_1_, whereas ΔNrf2, ΔHO-1 and ΔGCLC were found to be significant predictors of ΔFEV_1_. On the whole R^2^ was 0.895 and R^2^ predicted 0.881 (Table 2) and baseline FEV_1_, age, sex, smoking status, pack-year, BMI and follow-up time accounted for 15,6% of ΔFEV_1_ variance.

**Table 2:** Hierarchical stepwise linear regression considering FEV_1_% variation as dependent variable and Nrf2, HO-1 and GCLC mRNA, plasma GSH, plasma 8-iso % variation and baseline smoking history (pack-year) as independent variables in COPD group (*n*.33)

Independent variable	Standardized Beta coefficients	*P* value	R^2^	Predicted R^2^
FEV_1_% variation				
Nr2 mRNA (% variation)	0.405	0.001		
HO-1 mRNA (% variation)	0.364	0.006		
GCLC mRNA (% variation)	0.322	0.015		
plasma 8-iso (% variation)	0.096	0.315		
plasma GSH (% variation)	0.074	0.403		
pack-year	0.059	0.477		
			0.895	0.881

Finally the association between the absolute values of the independent variables of interest and FEV_1_ was investigated by means of a linear mixed model (Table 3). After adjusting for potential confounders, the absolute value of Nrf2 mRNA was significantly associated with FEV_1_, in particular for an increase in one unit of Nrf2 mRNA, FEV_1_ increased by about 8.31% (95%CI, 4.72;11.91%). The absolute values of HO-1 mRNA, GCLC mRNA, 8-iso and GSH plasma concentrations were not associated with FEV_1_.

**Table 3:** Fixed effect estimators for the 2-level random intercept linear model with level 1 units (measurements/visits) nested into level 2 units (*n* = 33 subjects with COPD), with FEV_1_% predicted as the dependent variable and Nrf2, HO-1 and GCLC mRNA, plasma GSH, plasma 8-iso as independent variables (fixed effects)

Dependent variables	Beta Coefficients	*P*-value	95%CI
Nrf2/beta actin mRNA	8.31	< 0.001	4.72;11.91
HO-1/beta actin mRNA	1.84	0.441	−2.84;6.53
GCLC/beta actin mRNA	6.40	0.118	−1.63;14.44
plasma GSH (μmol/L)	0.03	0.785	−0.18;0.24
plasma 8-iso (pg/ml)	−0.01	0.284	−0.02;0.01

Legend: The model was adjusted also for age, sex, smoking status, smoking history (pack year), BMI and follow-up time

## Discussion

COPD is a slowly progressive disease characterized by irreversible airflow obstruction in which continuous cigarette smoking-induced oxidative stress and inflammation are identified as the major pathogenetic factors [6, 7]. Available body of information indicates that cigarette smoking discontinuation is helpful in reducing mortality and delaying, but not blocking the rate of lung function decline in patients with mild COPD [23], so that it is possible to hypothesize that oxidative stress and inflammation may persist even after cigarette smoking cessation [5, 24]. Studies carried out in the last few years on this topic have included mainly mild-severe COPD patients with different cigarette smoking exposure [5, 25, 26]. In particular Malhotra et al. [26] found an increased oxidative stress in pulmonary tissues of both smokers and ex-smokers severe COPD patients. In these patients, abnormally high oxidative stress was not associated with an up-regulation of Nrf2/ARE genes in lung tissue that have been shown to protect lung against oxidative stress [25, 26]. In the present study we show that at baseline oxidative stress and inflammation were higher in mild moderate COPD patients than in no-COPD subjects despite similar prevalence of active cigarette smoking and of the other cardiovascular risk factors. Rather unexpectedly, but in line with our previous results [14], high oxidative stress was associated with an up-regulation of Nrf2/ARE gene expression in PBMCs derived from mild-moderate COPD patients. Taking together our results with those of Malhotra [26] it seems conceivable that the stage of the disease (or better the grade of bronchial obstruction) may be one of the main determinant of Nrf2 response to oxidative stress in COPD patients. However it has to be pointed out that the majority of data available so far on oxidative stress and Nrf2/ARE genes comes from airway cells or lung tissues [11, 27, 28], while in this study we considered circulating markers of oxidative stress and inflammation and mRNA derived from PBMCs of the subjects participating to the study. In this context, previous studies indicate that PBMC gene expression may be a promising noninvasive useful alternative to biopsy or invasive procedures especially at an early stage of the disease and a possible expression of systemic involvement of COPD [29, 30]. Taken together, our results at baseline strongly suggest that our mild moderate COPD subjects can still rise Nrf2/ARE expression in response to the intracellular oxidative stress. This capacity to counterbalance oxidative stress by increasing Nrf2 pathway is a new acquisition that could imply several consequences since many studies carried out in animal models of COPD and COPD patients evidently show that Nrf2 is a key player of COPD susceptibility [26, 29–33].

The results of this study also demonstrate that at baseline and after the period of follow-up plasma CRP and WBCs were higher in COPD patients than in no-COPD subjects. The increase of this systemic markers of inflammation agrees with previous studies suggesting that inflammation is a peculiarity of COPD patients regardless of active cigarette smoking [34, 35]. Even if the origin of inflammation may be multifactorial, it is likely that the persistent oxidative stress plays a key role since the oxidant-antioxidant imbalance has been identified as one of the determinants that eventually causes lung inflammation [6].

In this study we also demonstrate that after the period of follow-up there was a decline of FEV_1_ in both groups although it was much greater in COPD patients than in no-COPD subjects. These results are in line with a previous review [16] and a recent study amongst a UK primary care COPD population, showing that FEV_1_ decline was faster in current smokers and COPD patients with milder airflow obstruction [36]. Interestingly we found that oxidative stress, as evaluated by circulating levels of 8-iso was even higher at the end of follow-up than at baseline in COPD patients while it did not change in no-COPD subjects. Furthermore our results also show that GSH significantly declined after the follow-up indicating that antioxidant defenses were lower than at baseline in COPD patients but not in no-COPD subjects. To our knowledge, this is the first demonstration of an oxidative-antioxidative imbalance that worsened after a long period of time regardless of active cigarette smoking in COPD patients. After the follow-up also CRP plasma concentrations and WBCs were higher than the values detected at baseline only in COPD group, supporting the idea that oxidative stress and inflammation may be strictly correlated in COPD patients [6].

Another very important and peculiar result of this study is that after the period of follow-up there was a significant down-regulation of the genes correlated with antioxidant defenses in COPD patients. Nrf2, HO-1 and GCLC mRNA expression resulted significantly lower than at baseline and the values were similar to those found in no-COPD subjects. It is likely therefore that the increase in antioxidant defenses that we have found at baseline and previously in mild-moderate COPD patients [14] is a peculiarity of the early stages of the disease and that over time antioxidant defenses fail. We have previously demonstrated that active tobacco smoking has a profound effect on oxidative stress and on Nrf2/ARE gene expression in young healthy people [15]. Recently, however, we showed that oxidative stress and Nrf2/ARE gene expression response were higher in mild/moderate COPD patients categorized as no or past-smokers [14], suggesting that at least in these COPD patients oxidative stress and Nrf2 response are not strictly related to active smoking. The reason why in this study Nrf2/ARE genes are no longer expressed in spite of considerable oxidative stress remains to be elucidated. Previous studies on this topic indicated that the decrease of Nrf2 in alveolar macrophages and lung tissues of patients with emphysema was due to an increase of Bach-1 and Kelch-like ECH-associated protein 1 [25] or a loss of Nrf2 protein stability [26]. In particular a reduction in the stabilizing protein DJ-1 has been associated with lower Nrf2 protein stability, increased Nrf2 degradation, reduced Nrf2-dependent antioxidant responses and persistent oxidative stress in the lungs of COPD patients [26]. Although the key mechanisms for diminished levels of DJ-1 in patients with COPD are unclear, earlier reports have indicated that DJ-1 is modified by oxidative stress [26]. DJ-1 is a redox-responsive protein that is oxidatively modified, made inactive and degraded via proteasomal degradation by cigarette smoking in airway epithelial cells [28] and over time in humans [37]. So, on the basis of these considerations we are tempting to speculate that the continuous oxidative stress and its worsening after the follow-up has reached the threshold of oxidation after which DJ-1 is degraded thus blocking the Nrf2-dependent oxidative defenses. Of course on the basis of the present results we cannot draw any definite conclusion on this specific topic and further studies are needed to support this hypothesis.

Very interestingly the results of this study also indicate that after the period of follow-up there was a significant relationship between the variation FEV_1_ and the variations of circulating parameters of oxidative stress and Nrf2/ARE gene expression in COPD patients. By using a hierarchical stepwise multiple linear regression ΔNrf2, GCLC and ΔHO-1 were found to be significant predictors of ΔFEV1, explaining 89,5% of its variance. To our knowledge, this is the first evidence that the decline of oxidative defenses and in particular of Nrf2/ARE genes after a relatively long period of observation may be one of the determinants of FEV_1_ decline in COPD patients. Furthermore, the fact that, by using mixed model analysis the absolute value of Nrf2 mRNA was significantly associated with FEV_1_ after adjusting for potential confounders, furtherly supports this view. Of course it has to be underlined that this relationship between Nrf2 mRNA and the outcome FEV_1_ should be interpreted in a different perspective since it estimates the association between the absolute level of the covariates and the outcome. Currently we cannot definitely indicate how the reduction of antioxidant defenses may affect lung function. Nevertheless it has been shown that Nrf2 has protective effects through the transcriptional activation not only of antioxidant but also of antiprotease genes in alveolar macrophages, which is attenuated in the lungs of Nrf2-deficient mice [38, 39]. Consistent with these findings, a cohort study on the relationship between polymorphisms of the Nrf2 gene and limitations of airflow in smokers also indicates that impaired Nrf2 may contribute to the development of COPD owing to excessive oxidant burden and apoptosis in the lungs [26]. Furthermore there are data showing that Nrf2 plays an important role in limiting NF-kB activation and cytokine overexpression in lung tissue thereby blocking inflammation [40]. Finally, Nrf2 may have an influence on the infection-related acute exacerbations and therapeutic responses to corticosteroids in COPD. In this context, a recent study revealed that deficit in Nrf2 may play an essential role in steroid resistance via histone deacetylase 2 (HDAC2) level and deacetylase activity repression: the recruitment of HDAC2 is important in mediating the anti-inflammatory activities of glucocorticoids by its interaction with promoters of proinflammatory genes [41].

## Conclusions

The results of this study show that at baseline oxidative stress, inflammation and the antioxidant Nrf2/ARE gene expression were higher in mild-moderate COPD patients than in no-COPD subjects regardless of active cigarette smoking and the other cardiovascular risk factors. After the period of follow-up there was a faster decline of FEV_1_ in COPD patients than in no-COPD subjects. At the same time there was a further increment of oxidative stress in COPD patients which was not associated with the Nrf2/ARE pathway activation, indicating that the stage of the disease may be one of the main determinant of Nrf2 response to oxidative stress in COPD patients. Finally the study indicates that the reduction of antioxidant defenses may be one of the determinants of FEV_1_ decline. Since there are currently no treatments that significantly reverse or slow the progression of COPD [6], the future possibility to counteract Nrf2 decline in COPD patients may help in reducing the negative effects of the oxidative stress-induced progression of the disease.

It has to be underlined however that there were many missing data between the baseline period and the end of follow-up. Despite some of the missing data were lost at random, a substantial number of missing data were not. This is a limitation of the study and may weaken the population representativity.

## Data Availability

The datasets used and analysed during the current study are available from the corresponding author on reasonable request.

## Abbreviations

8-iso: 8 isoprostane
COPD: Chronic Obstructive Lung Disease
CRP: High sensitivity C-reactive protein
FEV_1_: Forced expiratory volume in 1st second
FVC: Forced vital capacity
GCLC: Glutamate-cysteine ligase catalytic
GOLD: Global Initiative for Chronic Obstructive Lung Disease
GSH: Glutathione
HO-1: Heme oxygenase-1
mRNA: Messenger RNA
Nrf2: Nuclear factor-E2-related factor 2
PBMCs: Peripheral blood mononuclear cells
PCR: Polymerase chain reaction
ROS: Reactive oxygen species
WBCs: White blood cells
