# Correction to: Oxidative stress and Nrf2 expression in peripheral blood mononuclear cells derived from COPD patients: an observational longitudinal study

**DOI:** 10.1186/s12931-020-01481-2

**Published:** 2020-08-12

**Authors:** A. M. Fratta Pasini, C. Stranieri, M. Ferrari, U. Garbin, L. Cazzoletti, C. Mozzini, F. Spelta, D. Peserico, L. Cominacini

**Affiliations:** 1 Department of Medicine, Section of General Medicine and Atherothrombotic and Degenerative Diseases, University of Verona, Verona, Italy; 2 Department of Medicine, Unit of Respiratory Diseases, Verona, Italy; 3 Department of Diagnostics and Public Health, Unit of Epidemiology & Medical Statistics, University of Verona, Verona, Italy

**Correction to: Respir Res 21, 37 (2020)**

**https://doi.org/10.1186/s12931-020-1292-7**

After publication of our article [1] it was brought to our attention that references 26 and 27 had been retracted in 2016.

Since these retracted studies are cited in Discussion to support the results of our study, the sentence on page 8 line 3, should be read: Studies carried out in the last few years on this topic have included mainly mild-severe COPD patients with different cigarette smoking exposure [5, 25]. In particular Goven et al. [25] found an increased oxidative stress in pulmonary tissues of severe COPD patients. In these patients, abnormally high oxidative stress was not associated with an up-regulation of Nrf2/ARE [25].

In the sentence on page 8, line 1 (column 2), reference 27 should not be considered and replaced with reference 25.

In the sentence on page 8, line 15 (column 2), reference 26 should not be considered.

On page 10, line 8 (column 2), “a loss of Nrf2 protein stability [26] should not be considered”.

The two sentences on page 10, from line 8 to line 16 (column 2) should be read “A further possibility is that there is a loss of Nrf2 protein stability due to overoxidation of DJ-1”.

The sentence from page 10 last line (column 2) to page 11, line 5, should be read “So, consistent with these findings, Nrf2 may contribute to the development of COPD owing to excessive oxidant burden and apoptosis in the lungs.”
